# Propagation properties of a non-linear mapping based on squaring in odd characteristic

**DOI:** 10.1007/s12095-024-00711-4

**Published:** 2024-04-26

**Authors:** Joan Daemen, Daniël Kuijsters, Silvia Mella, Denise Verbakel

**Affiliations:** Digital Security, Radboud University, Toernooiveld 212, Nijmegen, 6525 EC The Netherlands

**Keywords:** Non-linear layer, Squaring, Finite fields, 94A60, 06E30

## Abstract

Many modern cryptographic primitives for hashing and (authenticated) encryption make use of constructions that are instantiated with an iterated cryptographic permutation that operates on a fixed-width state consisting of an array of bits. Often, such permutations are the repeated application of a relatively simple round function consisting of a linear layer and a non-linear layer. These constructions do not require that the underlying function is a permutation and they can plausibly be based on a non-invertible transformation. Recently, Grassi proposed the use of non-invertible mappings operating on arrays of digits that are elements of a finite field of odd characteristic for so-called MPC-/FHE-/ZK-friendly symmetric cryptographic primitives. In this work, we consider a mapping that we call $\gamma$ that has a simple expression and is based on squaring. We discuss, for the first time, the differential and linear propagation properties of $\gamma$ and observe that these follow the same rules up to a relabeling of the digits. This is an intriguing property that, as far as we know, only exists for $\gamma$ and the binary mapping $\chi _{3}$ that is used in the cryptographic permutation Xoodoo. Moreover, we study the implications of its non-invertibility on differentials with zero output difference and on biases at the output of the $\gamma$ mapping and show that they are as small as they can possibly be.

## Introduction

The round functions in cryptographic permutations of the type Substitution-Permutation Networks (SPN) consist of a non-linear layer and a linear layer. These layers are chosen and combined so that there is no exploitable differential propagation from input to output or exploitable correlations between input and output when used in the context of a construction like the sponge or duplex construction [1], Farfalle [2] or Even-Mansour [3]. The relevant properties of these mappings over binary fields have been studied extensively, leading to solid designs. However, in the last years there has been a growing interest in similar functions operating on arrays of digits that are elements of a field of odd characteristic. For instance, Kölbl et al. designed a ternary cryptographic hash function called Troika [4]. Other examples are the symmetric primitives defined over $\mathbb {F}_{p}^{n}$ like MiMC [5], GMiMC [6], Poseidon [7], Ciminion [8], and many others. These are designed to be efficient in the context of Secure Multi-Party Computation (MPC), Fully Homomorphic Encryption (FHE), and Zero-Knowledge proofs (ZK).

There are interesting differences between fields $\mathbb {F}_{2^d}$ of characteristic 2 and those of odd characteristic that we will denote by $\mathbb {F}_{q}$. For instance, addition and subtraction are the same in $\mathbb {F}_{2^d}$, but this is not the case in $\mathbb {F}_{q}$. In $\mathbb {F}_{2^d}$, squaring is a linear operation, whereas in $\mathbb {F}_{q}$ squaring is a non-linear operation. In $\mathbb {F}_{2}$, correlations between input and output bits have values that are rational and range from $-1$ to 1, but in $\mathbb {F}_{p}$, correlations are complex numbers inside the closed unit disk.

This work investigates a mapping over $\mathbb {F}_{q}^{n}$ that was recently proposed by Grassi [9] and that we call $\gamma$. This is the mapping defined over $\mathbb {F}_{q}^{n}$ by $\gamma _i(x) = x_i + x_{i+1}^2$ for $i \in \mathbb {Z}/n\mathbb {Z}$ and for all $x \in \mathbb {F}_{q}^{n}$.

The paper is organized as follows. Section 2 deals with commonly used notation and conventions that we follow. In Section 3 we recall the basic notions from differential cryptanalysis. An overview of correlation analysis is presented in Section 4. In Section 5 we apply this existing theory to the squaring transformation and derive its DP and LP values. Based on the squaring transformation, we motivate the choice for $\gamma$ in Section 6. The main contribution of this paper lies in Sections 7 and 8, where we study the differential and linear propagation properties of $\gamma$, both in the forward and backward direction. Our results are useful in determining the maximum probabilities of differentials and differential trails over transformations making use of $\gamma$ in their round function, as in computer-assisted trail search [10]. Moreover, as the differential and linear propagation properties of $\gamma$ follow the same rules, our results are also useful to study the correlations of linear approximations and linear trails. In Section 9 we study the collision probability and bias of linear combinations of output digits of $\gamma$. Finally, we conclude in Section 10.

## Notation and conventions

We denote by $\mathbb {F}_{q}$ the finite field of *odd* characteristic *p*, i.e., *q* is equal to $p^d$ for some odd prime *p* and positive integer $d > 0$. Let $\mathbb {F}_{q}^{n}$ be the vector space of dimension *n* over the finite field $\mathbb {F}_{q}$. Given two vectors $x,y \in \mathbb {F}_{q}^{n}$, we denote their vector subtraction by $x - y$, i.e., $x - y = x + (-1)y$. A vector $x \in \mathbb {F}_{q}^{n}$ is indexed by the set $\mathbb {Z}/n\mathbb {Z}$. We denote its *i*th coordinate by $x_i$ and call it a *digit*. The dot product between *x* and *y* is defined as $x^{\top } y = \sum _{i=0}^{n-1} x_iy_i$. We write $e_i$ for the vector with all digits equal to 0, except for the digit that is indexed by *i*, which is equal to 1. The linear span of a set of vectors $S \subseteq \mathbb {F}_{q}^{n}$ is denoted by $\text {Span}(S)$. A digit is said to be *active* if it is non-zero. The Hamming weight $\textrm{HW}(x)$ of a vector $x \in \mathbb {F}_{q}^{n}$ is the number of active digits in the vector.

Let $z \in \mathbb {C}$ be a complex number. We denote its absolute value as |*z*|. We write $\overline{z}$ for its complex conjugate.

Let *F* be a field, then we write $F^{*}$ for its multiplicative group $F \setminus \{0\}$.

## Differential analysis

First published by Biham and Shamir in [11], *differential cryptanalysis* is a chosen-plaintext attack that exploits the non-uniformity of the distribution of differences at the output of a transformation when it is applied to pairs of inputs with a fixed difference.

Any successful theory of cryptanalysis needs to address the problem of secret key translation. Differential cryptanalysis deals with this problem by considering differences, which are invariant under translation. Let $x \in \mathbb {F}_{q}^{n}$ and $x^* \in \mathbb {F}_{q}^{n}$ be inputs of a transformation $\alpha :\mathbb {F}_{q}^{n} \rightarrow \mathbb {F}_{q}^{n}$ and let their difference be $a = x^* - x$. Likewise, let $y \in \mathbb {F}_{q}^{n}$ and $y^* \in \mathbb {F}_{q}^{n}$ be outputs of $\alpha$ and let their difference be $b = y^* - y$. The (ordered) pair $(a, b) \in \mathbb {F}_{q}^{n} \times \mathbb {F}_{q}^{n}$ containing the input and output difference is called a *differential* over $\alpha$. The differential (0, 0) is called *trivial*. The *differential probability (DP)* of a differential (*a*, *b*) over the transformation $\alpha$ is defined as

$$\begin{aligned} \textrm{DP}_\alpha (a,b) = q^{-n}\left| \{x \in \mathbb {F}_{q}^{n} : \alpha (x+a) - \alpha (x) = b\}\right| \,. \end{aligned}$$

If $\textrm{DP}_\alpha (a,b)>0$, we say that *a* and *b* are *compatible* differences over $\alpha$. For compatible differences *a* and *b*, we define the weight of a differential (*a*, *b*) over $\alpha$ as

$$\begin{aligned} \textrm{w}_\alpha (a,b) = -\log _{q}(\textrm{DP}_\alpha (a,b)) \,. \end{aligned}$$

A non-trivial differential (*a*, *b*) over $\alpha$ can only lead to a distinguisher if $\textrm{DP}_\alpha (a, b)$ differs significantly from $q^{-n}$, which is the expected DP of any non-trivial differential over a randomly selected transformation of $\mathbb {F}_{q}^{n}$.

## Correlation analysis

Correlation analysis of cryptographic primitives effectively is Fourier analysis on finite abelian groups. As such, the theory is well-understood and this section serves as a recap. The ideas that we present here are based on the works of Daemen [12], Baignères et al. [13], and Daemen and Rijmen [14]. Many of the proofs can be found in the book by Hou [15].

### Characters

Let $(G,+)$ be a finite abelian group and let *e* be the (finite) exponent of *G*, i.e., the smallest integer *n* such that $na = 0$ for all $a \in G$.

A *character* of *G* is a homomorphism from *G* into the subgroup of $\mathbb {C}^{*}$ consisting of the *e*th roots of unity. The set of characters of *G* is denoted by $\hat{G}$ and it forms a group under the multiplication defined by $(\chi \chi ')(a) = \chi (a)\chi '(a)$ for all $a \in G$ and $\chi , \chi ' \in \hat{G}$. The groups *G* and $\hat{G}$ are isomorphic, but this isomorphism is not canonical.

For a fixed isomorphism between *G* and $\hat{G}$ and for each $a \in G$, we write $\chi _a$ for the image of *a* under this isomorphism. In particular, the character $\chi _0$ that is defined by $\chi _0(a) = 1$ for all $a \in G$ is called the *trivial character* and it is the identity element of the group $\hat{G}$.

Now, let $(G, +,\cdot )$ be the commutative ring that is obtained by introducing a multiplicative structure on *G*. This is always possible by the fundamental theorem of finite abelian groups. A character $\chi \in \hat{G}$ is called a *generating character* for *G* if $\chi _a(b) = \chi (ab)$ for all $a, b \in G$. If a commutative ring has a generating character for its additive group, then $\chi _a(b) = \chi (ab) = \chi (ba) = \chi _b(a)$. In the case that *G* is the direct sum of *n* copies of a commutative ring *R* and if *R* has a generating character, say $\phi$, then we obtain a generating character $\chi$ for *G* by setting $\chi (a_1, \ldots , a_n) = \phi (a_1) \cdots \phi (a_n)$. It holds that $\chi _{a}(b) = \chi (ab) = \phi (a^{\top } b)$, where the multiplication in *G* is defined component-wise.

As an example, consider *G* equal to $\mathbb {F}_{q}$ and put $\omega = e^{2 \pi i / p}$. Let $\textrm{Tr}:\mathbb {F}_{q} \rightarrow \mathbb {F}_{p}$ be the absolute trace function that is defined by $\textrm{Tr}(x) = \sum _{i=0}^{d-1} x^{p^i}$. This is a linear mapping. Each $u \in \mathbb {F}_{q}$ defines a generating character $\chi _u$ for $\mathbb {F}_{q}$ that is defined by

$$\begin{aligned} \chi _u(x) = \omega ^{\textrm{Tr}(ux)}, \qquad x \in \mathbb {F}_{q} \,. \end{aligned}$$

As a second example, consider *G* equal to $\mathbb {F}_{q}^{n}$, which is a direct sum of *n* copies of $\mathbb {F}_{q}$. Hence, each $u \in \mathbb {F}_{q}^{n}$ gives a generating character $\chi _u$ for $\mathbb {F}_{q}^{n}$ that is defined by

$$\begin{aligned} \chi _u(x) = \omega ^{\textrm{Tr}(u^{\top }x)}, \qquad x \in \mathbb {F}_{q}^{n} \,. \end{aligned}$$

### The Fourier transform

Consider the set $L^2(G)$ of functions $f :G \rightarrow \mathbb {C}$. Fix an ordering of the elements of *G*, e.g., $G = \{a_0, \ldots , a_{n-1}\}$. We write $\upsilon _f = (f(a_0), \ldots , f(a_{n-1}))$ for the finite sequence of the output values of *f*. By identifying a function *f* with the vector $\upsilon _f \in \mathbb {C}^{|G|}$, $L^2(G)$ can be seen as a finite-dimensional complex inner product space with inner product

$$\begin{aligned} \langle f, g \rangle = \sum _{a \in G} f(a)\overline{g(a)}, \qquad f, g \in L^2(G) \,. \end{aligned}$$

For any $f \in L^2(G)$, the inner product induces a norm by setting

$$\begin{aligned} \Vert f \Vert = \langle f, f \rangle ^{\frac{1}{2}} \,. \end{aligned}$$

The standard basis of $L^2(G)$ is formed by the set of Dirac delta functions $\{ \delta _a \in L^2(G) : a \in G\}$, which are defined by

$$\begin{aligned} \delta _a(b) = {\left\{ \begin{array}{ll} 1 &  \text { if } a = b \,, \\ 0 &  \text { if } a \ne b \,. \end{array}\right. } \end{aligned}$$

In the context of correlation analysis, the solution to the problem of secret key translation lies in changing the basis of $L^2(G)$ to the set of characters of *G*. For any $a, b \in G$, the corresponding characters satisfy $\langle \chi _a, \chi _b \rangle = |G|\delta _a(b)$. By normalizing the characters, we obtain an orthonormal basis

$$\begin{aligned} \Phi _G&= \{ \phi _a : a\in G\} \,, \end{aligned}$$

where $\phi _a = |G|^{-\frac{1}{2}}\chi _a$. By projecting *f* onto $\Phi _G$, we find that

$$\begin{aligned} f = \sum _{a \in G} \langle f, \phi _a \rangle \phi _a \,. \end{aligned}$$

The operator $\mathcal {F} :L^2(G) \rightarrow L^2(G)$ that is defined by $\mathcal {F}(f)(a) = \langle f, \phi _a \rangle$ for all $a \in G$ is called the *Fourier transform*. By identifying a function *f* with $\upsilon _f$, the Fourier transform is best described as assigning to *f* its coordinates in the normalized character basis. The *Plancherel theorem* asserts that the Fourier transform is unitary, i.e., we have

$$\begin{aligned} \langle \mathcal {F}(f), \mathcal {F}(g) \rangle = \langle f, g \rangle , \qquad f, g \in L^2(G) \,. \end{aligned}$$

Let us return to the question of how to address the problem of secret key translation. Let $b \in G$. We define the translation operator $T_b :L^2(G) \rightarrow L^2(G)$ by $(T_b f)(a) = f(a + b)$ for all $a \in G$. Moreover, we define the modulation operator $M_b :L^2(G) \rightarrow L^2(G)$ by $(M_b f)(a) = \phi _b(a)f(a)$ for all $a \in G$. The big insight is that translation turns into modulation when changing from the standard basis to the normalized character basis, i.e.,

$$\begin{aligned} T_b = \mathcal {F}^{-1} \circ M_b \circ \mathcal {F}, \qquad b \in G \,. \end{aligned}$$

Let *H* be a finite abelian group and let $F :G \rightarrow H$ be a mapping between *G* and *H*. We want a representation of *F* in $L^2(G)$. To that end, let $\chi$ be any character of *H*. We take as representation the function $\chi \circ F \in L^2(G)$.

### Correlation

We now specialize to the case that *G* and *H* are each equal to the vector space $\mathbb {F}_{q}^{n}$ over the finite field $\mathbb {F}_{q}$.

Let $\alpha :\mathbb {F}_{q}^{n} \rightarrow \mathbb {F}_{q}^{n}$ be a transformation of $\mathbb {F}_{q}^{n}$. We consider pairs $(u, v) \in \mathbb {F}_{q}^{n} \times \mathbb {F}_{q}^{n}$ that we call *linear approximations* of $\alpha$. We refer to *u* as the *output mask* and to *v* as the *input mask*. The linear approximation (0, 0) is called *trivial*. The *correlation* of the linear approximation is defined as

$$\begin{aligned} \textrm{C}_{\alpha }(u, v) = q^{-\frac{n}{2}} \mathcal {F}(\chi _u \circ \alpha )(v) \,. \end{aligned}$$

We call the masks *u* and *v* *compatible* over $\alpha$ if $\textrm{C}_{\alpha }(u, v)$ is nonzero. In general, correlations are complex numbers. The *linear potential (LP)* is a real number and related to a correlation by

$$\begin{aligned} \textrm{LP}_{\alpha }(u,v) = \textrm{C}_{\alpha }(u,v) \overline{\textrm{C}_{\alpha }(u,v)} \,. \end{aligned}$$

If *u* and *v* are compatible over $\alpha$, then we can define the *weight* of the linear approximation (*u*, *v*) as

$$\begin{aligned} \textrm{w}_\alpha (u,v) = -\log _q(\textrm{LP}_\alpha (u,v)) \,. \end{aligned}$$

## The squaring transformation

The squaring transformation $\beta :\mathbb {F}_{q} \rightarrow \mathbb {F}_{q}$ is defined by $x \mapsto x^2$ for all $x \in \mathbb {F}_{q}$. Because we study the case of odd characteristic, $\beta$ is non-linear. We show that $\beta$ has the property that the maximal DP over all non-trivial differentials is $q^{-1}$, which is the smallest possible value. A similar property holds for the maximal LP over all non-trivial linear approximations. In other words, we show that $\beta$ is a *bent* polynomial [16]. Note that this is an improvement from the case of characteristic 2, for which these values are both equal to $2q^{-1}$ and are obtained by, respectively, almost perfect nonlinear and bent functions [17].

First, by applying Theorem 5.33 from [18], we obtain that the correlation of any linear approximation $(u, v) \in \mathbb {F}_{q} \times \mathbb {F}_{q}$ with $u \ne 0$ of $\beta$ is equal to

$$\begin{aligned} \textrm{C}_{\beta }(u,v)&= q^{-\frac{1}{2}}\mathcal {F}(\chi _u \circ \beta )(v) \\&= q^{-1}\sum _{x \in \mathbb {F}_{q}}\chi _1(ux^2 - vx) \\&= {\left\{ \begin{array}{ll} q^{-\frac{1}{2}}(-1)^{d-1} \chi _1(-v^2 (4u)^{-1}) \eta (u) &  \text {if} \ p \equiv 1 \pmod 4 \,, \\ q^{-\frac{1}{2}}(-1)^{d-1} i^d \chi _1(-v^2 (4u)^{-1}) \eta (u) &  \text {if} \ p \equiv 3 \pmod 4 \,, \end{array}\right. } \end{aligned}$$

where $\eta (u) = 1$ if *u* is a square in $\mathbb {F}_{q}$ and $-1$ otherwise. It follows that for all $u, v \in \mathbb {F}_{q}$ with $u \ne 0$ we have $\textrm{LP}_\beta (u,v) = q^{-1}$. In particular, choosing *v* equal to zero shows that any linear combination of output digits of $\beta$ is imbalanced, i.e., the distribution of this linear combination is non-uniform. If *u* is 0, then for all nonzero $v \in \mathbb {F}_{q}$ we have $\textrm{LP}_\beta (0, v) = 0$, and $\textrm{LP}_\beta (0, 0) = 1$.

Second, consider the equation that relates the input $x \in \mathbb {F}_{q}$, the input difference $a \in \mathbb {F}_{q}$, and the output difference $b \in \mathbb {F}_{q}$, i.e.,

$$\begin{aligned} b&= \beta (x + a) - \beta (x) \\&= (x+a)^2 - x^2 \\&= x^2 +2ax + a^2 - x^2 \\&= 2ax+a^2 \,. \end{aligned}$$

Assuming that $a \ne 0$ and because the characteristic of $\mathbb {F}_{q}$ is odd, we can solve for *x* to find that $x = (2a)^{-1}(b - a^2)$. Hence, there is exactly one solution to this equation. Dividing by the domain size, *q*, then shows that $\textrm{DP}_\beta (a, b) = q^{-1}$. In particular, any nonzero input difference can propagate to a zero output difference. If *a* is 0, then for all nonzero $b \in \mathbb {F}_{q}$, we have $\textrm{DP}_\beta (0, b) = 0$ and $\textrm{DP}_\beta (0, 0) = 1$.

We summarize these properties to make the symmetry between the differential and linear case apparent:

- For all $a,u \in (\mathbb {F}_{q})^*$ and $b,v \in \mathbb {F}_{q}$, we have $\textrm{DP}_\beta (a,b) = \textrm{LP}_\beta (u,v) = q^{-1}$;
- For all $b,v \in (\mathbb {F}_{q})^*$, we have $\textrm{DP}_\beta (0,b) = \textrm{LP}_\beta (0, v) = 0$;
- We have $$\textrm{DP}_\beta (0,0) = \textrm{LP}_\beta (0,0) = 1$$.

## The $\gamma$ mapping

Some modern block cipher modes, like GCM [19], CTR and OFB [20], do not use the inverse block cipher. Similarly, constructions like sponge [21], duplex [1], and Farfalle [2], which are generally based on permutations, do not use their inverse. Therefore, in such constructions permutations can be replaced by transformations. An example is the GLUON family of lightweight hash functions [22], which makes use of the sponge construction on top of a non-invertible map.

A cryptographic transformation can be used as long as collisions and imbalances in the output cannot be exploited. This can be tackled by either ensuring that such imbalance is very small or by limiting the attacker’s access to the input and output of the transformation by construction. For instance, in the sponge and duplex constructions the attacker has control of only the outer part of the state and not of its inner part. Therefore, if a collision requires a difference in the inner part of the state at the input of the transformation, the attacker cannot inject it with input messages. Similarly, the attacker has no visibility of the inner bits or digits of any output mask. As another example, whitening keys can be added at input and output, like in Farfalle [2], Even-Mansour [3], and Elephant [23].

We consider the problem of building a non-invertible mapping based on squaring that can be used as non-linear layer in the round function of cryptographic transformations. When such transformations are used in constructions that are usually instantiated with permutations, the non-invertibility of the mapping should be difficult to exploit.

By definition, such a non-linear layer has pairs of distinct inputs that are mapped to the same output, i.e., collisions. A naive idea would be to apply $\beta$ to each digit of the state independently. The problem with this approach is that each collision for $\beta$ is trivially extended to a collision for the entire non-linear layer, giving rise to differentials with $\textrm{DP}$ as high as $q^{-1}$. They are easy to exploit as the adversary needs access to only a single input digit to generate a local collision. Similarly, any bias in the output of $\beta$ is trivially present in the output of the non-linear layer, giving rise to linear approximations with $\textrm{LP}$ as high as $q^{-1}$. They are easy to exploit as the adversary needs access to only a single output digit to exploit them. The measure of both is inversely proportional to the order of the field. Hence, unless the order of the field is very large, this leads to unacceptable weaknesses in the cryptographic transformation.

Compared to the above, the non-linear layer in the round function of a cryptographic transformation should have lower $\textrm{DP}$ and $\textrm{LP}$ and there should not exist local properties that can be extended to global properties. We achieve this by making the $\textrm{DP}$ of differentials of the form (*a*, 0) and the $\textrm{LP}$ of linear approximations of the form (*u*, 0) small, i.e., equal to the inverse of the domain size. Moreover, any differential over or linear approximation of the non-linear layer requires access to every digit of the state.

The work by Grassi [9] presents an analysis of a number of mappings based on $\beta$ that minimize the probability of a collision in their output. We consider one of these mappings and call it $\gamma$. Concretely, the mapping $\gamma :\mathbb {F}_{q}^{n} \rightarrow \mathbb {F}_{q}^{n}$ is defined, for all $x \in \mathbb {F}_{q}^{n}$, by

$$\begin{aligned} \gamma _i(x) = x_i + x^2_{i+1}, \qquad i \in \mathbb {Z}/n\mathbb {Z} \,. \end{aligned}$$

The remainder of this text is concerned with an analysis of the differential and linear propagation properties of $\gamma$.

## Differential propagation properties of $\gamma$

Let $(a, b) \in \mathbb {F}_{q}^{n} \times \mathbb {F}_{q}^{n}$ be a differential over $\gamma$ and let $x \in \mathbb {F}_{q}^{n}$ be an input of $\gamma$. The equations that relate the input difference *a* and the output difference *b* are of the form

1 $$\begin{aligned} b_i = a_i + a_{i+1}^2 + 2a_{i+1}x_{i+1}, \qquad i \in \mathbb {Z}/n\mathbb {Z} \,. \end{aligned}$$

We consider two cases in the analysis of these equations. In the first case, we fix the input difference *a* and give a description of the set of compatible output differences *b*. From this, we are able to deduce that $\textrm{DP}_\gamma (a, b)$ depends only on *a* and whether *b* is compatible with *a* or not.

In the second, reverse case, we fix the output difference *b* and present an algorithm for the computation of the set of compatible input differences *a*. We then derive an expression of the so-called minimum reverse weight of this set. All these results can be directly applied to perform computer-aided trail search, as described in [10], in cryptographic transformations instantiated with $\gamma$ as the non-linear layer.

### Forward propagation from a given input difference

We observe that for an input difference *a*, the equations of (1) are linear in the digits of *x*. We make this explicit by writing them as a matrix equation of the form

$$\begin{aligned} \begin{pmatrix} b_0\\ b_1\\ b_2\\ \vdots \\ b_{n-2}\\ b_{n-1} \end{pmatrix}&= \begin{pmatrix} a_0 + a_1^2 \\ a_1 + a_2^2 \\ a_2 + a_3^2 \\ \vdots \\ a_{n-2} + a_{n-1}^2 \\ a_{n-1} + a_0^2 \end{pmatrix} + \begin{pmatrix} 0 &  2a_1 &  0 &  0 &  \cdots &  0 &  0 \\ 0 &  0 &  2a_2 &  0 &  \cdots &  0 &  0\\ 0 &  0 &  0 &  2a_3 &  \cdots &  0 &  0 \\ \vdots \\ 0 &  0 &  0 &  0 &  \cdots &  0 &  2a_{n-1} \\ 2a_0 &  0 &  0 &  0 &  \cdots &  0 &  0 \\ \end{pmatrix} \begin{pmatrix} x_0\\ x_1\\ x_2\\ \vdots \\ x_{n-2}\\ x_{n-1} \end{pmatrix} \,. \end{aligned}$$

Hence, the set of compatible output vectors *b*, which we denote by $\mathcal {A}(a)$, forms an affine subspace of $\mathbb {F}_{q}^{n}$. By affine subspace we mean the following. Let *W* be a linear subspace of $\mathbb {F}_{q}^{n}$ and let $u \in \mathbb {F}_{q}^{n}$. The coset $u + W = \{u + w : w \in W\}$ is called an affine subspace of $\mathbb {F}_{q}^{n}$ and *u* is called an offset. The affine subspace $\mathcal {A}(a)$ can be described by

$$\begin{aligned} \mathcal {A}(a) = \gamma (a) + \text {Span}\{2a_ie_{i-1} : i \in \mathbb {Z}/n\mathbb {Z}\} \,. \end{aligned}$$

Two cosets $u + W$ and $v + W$ are equal if and only if $u - v \in W$. Therefore, we may add any linear combination of the basis vectors to the offset without it changing the affine subspace that is defined. Moreover, we may scale the basis vectors by any nonzero constant. Hence, a description of $\mathcal {A}(a)$ in which the offset has minimal Hamming weight is given by

$$\begin{aligned} \mathcal {A}(a) = a' + \text {Span}\{e_{i} : i \in \mathbb {Z}/n\mathbb {Z} \text { and } a_{i+1} \ne 0 \} \,, \end{aligned}$$

where

$$\begin{aligned} a'_i = {\left\{ \begin{array}{ll} a_i &  \text { if } a_{i+1} = 0 \,, \\ 0 &  \text { if } a_{i+1} \ne 0 \,. \end{array}\right. } \end{aligned}$$

Clearly, the dimension of $\mathcal {A}(a)$, which is defined as the dimension of the associated vector space, is equal to the Hamming weight of *a*.

We are now ready to give a complete characterization of the distribution of differentials over $\gamma$.

#### Proposition 1

Let $(a, b) \in \mathbb {F}_{q}^{n} \times \mathbb {F}_{q}^{n}$ be a differential over $\gamma$. Then *b* is compatible with *a*, i.e., $b \in \mathcal {A}(a)$, if and only if, for all $i \in \mathbb {Z}/n\mathbb {Z}$, we have $b_i = a_i$ or $a_{i+1} \ne 0$, in which case $b_i$ can take on any value. Hence,

$$\begin{aligned} \textrm{DP}_\gamma (a,b) = {\left\{ \begin{array}{ll} q^{-\textrm{HW}(a)} &  \text { if } b \in \mathcal {A}(a) \,, \\ 0 &  \text { if } b \notin \mathcal {A}(a) \,. \end{array}\right. } \end{aligned}$$

In other words, the DP of a valid differential, and thus its differential weight, is a constant that depends only on the input difference.

### Backward propagation from a given output difference

For a given output difference *b*, the compatible input differences do not form an affine space. However, we will show in this section how to efficiently generate all compatible input differences *a* with $\textrm{w}_\gamma (a,b) \le W$ for some weight limit *W*. To this end, we introduce the concept of compatible activity pattern. Given a vector $x \in \mathbb {F}_{q}^{n}$, its activity pattern $\widetilde{x}$ is a vector in $\mathbb {F}_{q}^{n}$ with $\widetilde{x}_i$ equal to 1 if $x_i \ne 0$ and 0 otherwise.

#### Definition 1

An activity pattern is compatible with *b* if there exists an input difference *a* that is compatible with *b* and for which $\widetilde{a}$ equals this activity pattern.

The generation of all compatible input differences is done in two phases: in the first phase, we generate the set of activity patterns compatible with *b*, and in the second phase, we determine for each compatible activity pattern the set of compatible input differences with that pattern.

We generate the compatible activity patterns in a recursive way in Algorithm 1, making use of the following proposition.

#### Proposition 2

Given a differential (*a*, *b*) over $\gamma$, the following properties hold:

1. if $a_{i} = 0$ and $b_{i-1} = 0$ then $a_{i-1} = 0$;
2. if $a_{i} = 0$ and $b_{i-1} \ne 0$ then $a_{i-1} \ne 0$.

#### Proof

The two properties immediately follow from (1). We have

$$\begin{aligned} b_{i-1} = a_{i-1} + a_i^2 + 2a_ix_i \,, \end{aligned}$$

and $$a_i=0$$ implies $$b_{i-1} = a_{i-1}$$. $$\square $$

In Algorithm 1, we start with an empty list of compatible activity patterns *L* (line 4) and a fully unspecified activity pattern $\widetilde{a}$ (line 6). Then we specify whether $\widetilde{a}_{n-1}=0$ (line 6) or 1 (line 7) (and thus whether $a_{n-1}$ is active or not) and based on this we incrementally determine the activity of all other digits from $a_{n-2}$ to $a_0$ using the procedure ${\textsf{buildActivity}}$. In this procedure, when $\widetilde{a}_i=0$ we use Proposition 2 to determine whether $\widetilde{a}_{i-1}=1$ or 0, otherwise we consider both possibilities (lines 16 and 17). When a compatible activity pattern is fully determined (i.e., when $i=0$ is reached) then it is added to list *L* (line 12).

**Algorithm 1:**
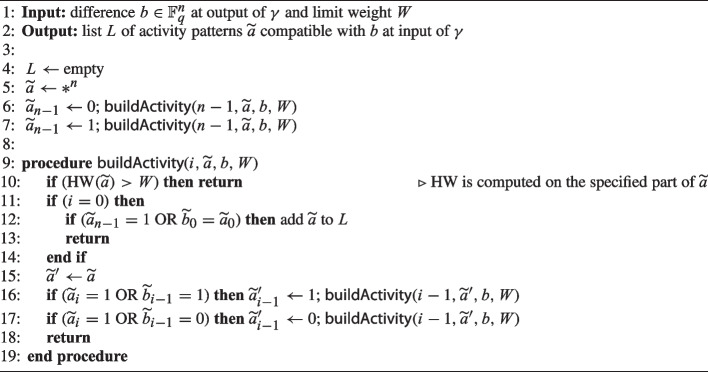
Generation of input activity patterns compatible with output difference *b*.

Given an output difference *b* and a compatible input activity pattern $\widetilde{a}$, we generate all compatible differences with activity $\widetilde{a}$ in Algorithm 2, making use of the following proposition.

#### Proposition 3

Given a differential (*a*, *b*) over $\gamma$, the following properties hold:

1. if $$\widetilde{a}_{i} = 0$$, then $$a_{i} = 0$$;
2. if $\widetilde{a}_{i} = 1$ and $\widetilde{a}_{i+1} = 0$, then $a_i = b_i$;
3. if $\widetilde{a}_{i} = 1$ and $\widetilde{a}_{i+1} = 1$, then $a_i$ can be any value in $\mathbb {F}_{q}$.

#### Proof

The first property follows from the definition of activity pattern. The other two properties immediately follow from (1). $\square$

In Algorithm 2, we start with an empty list of compatible input differences *L* (line 4) and a fully unspecified difference *a* (line 5). We use the symbol $*$ when the activity of a digit is unspecified. Then we incrementally determine the value of all digits from $a_0$ to $a_{n-1}$ using the procedure ${\textsf{buildDifference}}$. In this procedure, we use Proposition 3 to determine whether $a_i=1$ or 0 (lines 10-12 and 16-18). When a compatible difference is fully determined (i.e., when $i=n-1$ is reached) then it is added to list *L* (line 10-12).

**Algorithm 2:**
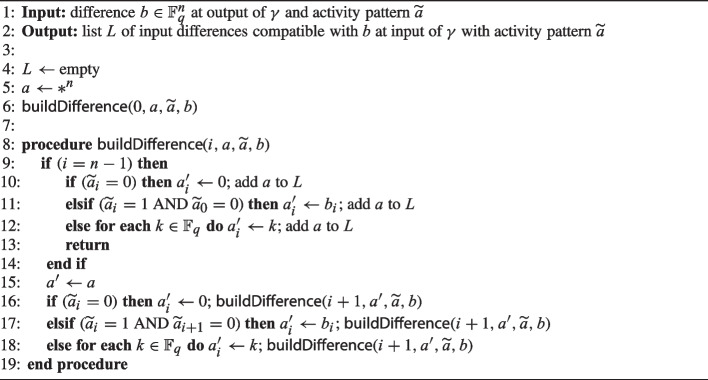
Generation of input differences compatible with output difference *b* and with activity pattern $\widetilde{a}$.

### Computing the minimum reverse weight of an output difference

Given an output difference *b*, let $\Omega (b) = \{a \in \mathbb {F}_{q}^{n} : \textrm{DP}_\gamma (a, b) > 0 \}$ be the set of input differences that are compatible with *b*. The differentials (*a*, *b*) over $\gamma$ with $a \in \Omega (b)$ can have different weights. Following [10], the *minimum reverse weight* of an output difference *b* is defined by

$$\begin{aligned} \textrm{w}^{\text {rev}}_\gamma (b)=\min _{a \in \Omega (b)} \textrm{w}_\gamma (a,b) \,. \end{aligned}$$

We notice that the minimum reverse weight of a difference *b* at the output of $\gamma$ is fully determined by its activity pattern and its compatible activity patterns with minimum Hamming weight. In particular, it can be computed as in the following Proposition, which uses the notion of *run*.

#### Definition 2

Given $x\in \mathbb {F}_{q}^n$, a run of length $\ell$ in *x* is a sequence of $\ell$ active digits preceded and followed by non-active digits, i.e., it satisfies $x_i, x_{i+1}, \ldots , x_{i+\ell -1} \ne 0$ and $x_{i-1} = x_{i+\ell } = 0$ for some $i \in \mathbb {Z}/n\mathbb {Z}$.

#### Proposition 4

Given a difference *b* at the output of $\gamma$ composed by *m* runs of lengths $\ell _j$, with $j = 0, \ldots , m-1$, then

$$\begin{aligned} \textrm{w}^{\text {rev}}_\gamma (b) = \sum _{j=0}^{m-1} \lceil \ell _j/2 \rceil \,. \end{aligned}$$

#### Proof

For a run starting in position *i* and of length $\ell$ in *b*, the digit $\widetilde{a}_{i+\ell -1}$ must be 1. There can be at most a single zero digit in between two active digits in the sequence $\widetilde{a}_i,\widetilde{a}_{i+1},\ldots , \widetilde{a}_{i+\ell -1}$. It follows that for each run of length $\ell$ in *b*, *a* has at least $\ell /2$ active digits if $\ell$ is even and $(\ell +1)/2$ if $\ell$ is odd. $\square$

## Linear propagation properties of $\gamma$

In this section we analyze the correlation properties of the mapping $\gamma$, starting with the correlation of linear approximations of $\gamma$.

### Proposition 5

Let $(u,v) \in \mathbb {F}_{q}^{n} \times \mathbb {F}_{q}^{n}$ be a linear approximation of $\gamma$. We have

$$\begin{aligned} \textrm{C}_{\gamma }(u,v) = \prod _{i=0}^{n-1} \textrm{C}_\beta (u_i - v_i,u_{i-1}) \,. \end{aligned}$$

### Proof

If we rewrite the correlation of a linear approximation of $\gamma$, we obtain

$$\begin{aligned} \textrm{C}_{\gamma }(u,v)&= q^{-n} \sum _{x \in \mathbb {F}_{q}^{n}} \omega ^{\textrm{Tr}\left( u^{\top } \gamma (x)-v^{\top } x \right) }\\&= q^{-n} \sum _{x \in \mathbb {F}_{q}^{n}} \omega ^{\textrm{Tr}\left( \sum _{i=0}^{n-1} u_i (x_i + x^2_{i+1}) - v_i x_i \right) } \\&= q^{-n} \sum _{x \in \mathbb {F}_{q}^{n}} \omega ^{\textrm{Tr}\left( \sum _{i=0}^{n-1} (u_i - v_i) x_i + u_{i-1} x^2_{i} \right) } \\&= q^{-n} \sum _{x \in \mathbb {F}_{q}^{n}} \omega ^{\sum _{i=0}^{n-1} \textrm{Tr}\left( (u_i - v_i) x_i + u_{i-1} x^2_{i} \right) } \\&= q^{-n} \sum _{x \in \mathbb {F}_{q}^{n}} \prod _{i=0}^{n-1} \omega ^{\textrm{Tr}\left( (u_i - v_i) x_i + u_{i-1} x^2_{i} \right) } \\&= \prod _{i=0}^{n-1} q^{-1} \sum _{y \in \mathbb {F}_{q}} \omega ^{\textrm{Tr}\left( (u_i - v_i) y + u_{i-1} y^2 \right) } \\&= \prod _{i=0}^{n-1} \textrm{C}_\beta (u_i - v_i,u_{i-1}) \,. \end{aligned}$$

$$\square $$

The resulting product from Proposition 5 is non-zero if each of the factors is non-zero. Note that the correlation is non-zero if $u_{i-1}$ is non-zero, as was discussed in Section 5. Additionally, if $u_{i-1}$ is non-zero, then $v_i - u_i$ has to be equal to zero to get a non-zero correlation. In this case it should thus hold that $v_i = u_i$. From this reasoning, we can give a complete characterization of the distribution of linear approximations of $\gamma$.

### Proposition 6

Let $(u,v) \in \mathbb {F}_{q}^{n} \times \mathbb {F}_{q}^{n}$ be a linear approximation of $\gamma$. Then *u* is compatible with *v*, if and only if, for all $i \in \mathbb {Z}/n\mathbb {Z}$, we have $v_i = u_i$ or $u_{i-1} \ne 0$, in which case $v_i$ can take on any value. Hence,

$$\begin{aligned} \textrm{LP}_\gamma (u,v) = {\left\{ \begin{array}{ll} q^{-\textrm{HW}(u)} &  \text {if v} \text { is compatible with u} \,, \\ 0 &  \text { if v} \text { is not compatible with u} \,. \end{array}\right. } \end{aligned}$$

Observe that Propositions 1 and 6 are very much alike. Indeed, propagation of differences and propagation of masks over $\gamma$ follow similar rules. First, output masks play the role of input differences and input masks that of output differences. Second, indices are reversed, i.e., index *i* in a mask corresponds to index $n-i-1$ in a difference, to account for this change in direction. The following proposition is an immediate consequence.

### Proposition 7

Let $\pi :\mathbb {F}_{q}^{n} \rightarrow \mathbb {F}_{q}^{n}$ be the mapping defined by $\pi _{i}(x) = x_{n-i-1} \text { for all } i \in \mathbb {Z}/n\mathbb {Z}$. Let (*u*, *v*) be a linear approximation of $\gamma$. We have

$$\begin{aligned} \textrm{LP}_\gamma (u, v) = \textrm{DP}_\gamma (\pi (u),\pi (v)) \,. \end{aligned}$$

From this, it follows that we can extend the results obtained in Section 7 to masks. For a given output mask $u \in \mathbb {F}_{q}^{n}$, we can build the affine subspace with dimension $\textrm{HW}(u)$ of compatible input masks over $\gamma$ as in Section 7.1. Moreover, for a given input mask $v \in \mathbb {F}_{q}^{n}$, the output activity patterns compatible with input masks over $\gamma$ can be found by applying Algorithm 1. Using the resulting activity pattern $\widetilde{a}$ and the input mask *v*, all compatible output masks *u* can be obtained as described in Algorithm 2. Note that there can be several compatible output masks *u* for a given input mask *v*. Among them, there will be one realizing the minimum value of $\textrm{w}(u,v)$. The *minimum reverse weight* of *v* is defined as

$$\begin{aligned} \textrm{w}^{\text {rev}}_\gamma (v)=\min _{u : \textrm{LP}_\gamma (u,v)>0} \textrm{w}_\gamma (u,v) \end{aligned}$$

and is determined by the decomposition of *v* in a sequence of runs, as explained in Section 7.3.

## On collision probability and bias

A collision in the output of $\gamma$ occurs when $\gamma$ maps a pair of different inputs $(x, y) \in \mathbb {F}_{q}^{n} \times \mathbb {F}_{q}^{n}$ to the same output value. Assuming randomly and uniformly selected pairs of inputs, the probability of a collision is given by

$$\begin{aligned} \textrm{CP}(\gamma ) = q^{-2n}|\{(x, y) \in \mathbb {F}_{q}^{n} \times \mathbb {F}_{q}^{n} : x \ne y \text { and } \gamma (x) = \gamma (y)\}| \,. \end{aligned}$$

Translating this into the language of differential analysis, we find that

$$\begin{aligned} \textrm{CP}(\gamma ) = q^{-n} \sum _{a \in \mathbb {F}_{q}^{n} \setminus \{0\}} \textrm{DP}_\gamma (a, 0) \,. \end{aligned}$$

### Proposition 8

Let $a \in \mathbb {F}_{q}^{n} \setminus \{0\}$. If (*a*, 0) is a differential with $\textrm{DP}_\gamma (a, 0) > 0$, then all digits of *a* are active and $\textrm{DP}_{\gamma }(a, 0) = q^{-n}$.

### Proof

Let $a \in \mathbb {F}_{q}^{n}\setminus \{0\}$ be such that $\textrm{DP}_\gamma (a, 0) > 0$. The input difference *a* is compatible with the output difference 0 if the latter is contained in the affine space *A*(*a*). This is the case if and only if $a_i \ne 0$ for $i \in \mathbb {Z}/n\mathbb {Z}$. Hence, $\textrm{DP}_\gamma (a, 0) = q^{-n}$ by Proposition 1. $\square$

Clearly, there are $(q-1)^n$ input differences *a* for which this property holds. Therefore, we find that

$$\begin{aligned} \textrm{CP}(\gamma ) = (q-1)^n q^{-2n} \,. \end{aligned}$$

Now, the collision probability of a function that is chosen randomly from the set of functions from $\mathbb {F}_{q}^{n}$ to $\mathbb {F}_{q}^{n}$ is equal to $q^{-n}$. Hence, the ratio between the collision probability of $\gamma$ and that of a random function is equal to $(1 - q^{-1})^n$. If the order of the field is large, then this quantity approximates 1.

By symmetry, we obtain a similar result for the bias of any linear combination of output digits of $\gamma$.

### Proposition 9

Let $u \in \mathbb {F}_{q}^{n} \setminus \{0\}$. If (*u*, 0) is a linear approximation with $\textrm{LP}_\gamma (u, 0) > 0$, then all digits of *u* are active and $\textrm{LP}_{\gamma }(u, 0) = q^{-n}$.

Clearly, if either *q* or *n* is large, then these quantities are very small and it becomes difficult to exploit them in practice.

## Conclusion

When searching for trails over an iterated cryptographic transformation as described in [10], a number of tools are required. These include an efficient method to compute the minimum reverse weight of a given difference (resp. mask), and an efficient method to build all compatible input differences (resp. output masks) over the non-linear layer for a given output difference (resp. input mask) and vice versa. In this work we provided such tools for a mapping based on squaring that can be used as non-linear layer in the construction of cryptographic transformations of $\mathbb {F}_{q}^{n}$. Interestingly, it turns out that for this mapping, masks and differences follow the same propagation rules. This means that for a cryptographic transformation that uses this mapping as the non-linear layer in its round function, one would need to only perform either differential or linear trail search while obtaining insights and bounds for both.
